# Seroprevalence of *Dirofilaria immitis* in traveling dogs: implications for transboundary pathogen movement

**DOI:** 10.1186/s13071-026-07540-9

**Published:** 2026-07-02

**Authors:** Theresa A. Quintana, Breck Aguinaga, Jessica Mitchell, Grace Schieferecke, Janine F. R. Seetahal, Jeba R. J. Jesudoss Chelladurai

**Affiliations:** 1 Department of Pathobiology, College of Veterinary Medicine, Auburn University, Auburn, AL USA; 2 Department of Diagnostic Medicine/Pathobiology, College of Veterinary Medicine, Kansas State University, Manhattan, KS USA; 3 Division of Biology, College of Arts and Science, Kansas State University, Manhattan, KS USA; 4 Kansas State Veterinary Diagnostic Laboratory, Manhattan, KS USA

**Keywords:** Dogs, Dirofilariasis, Enzyme-linked immunosorbent assay, Zoonoses, Travel

## Abstract

**Background:**

*Dirofilaria* spp. are parasitic filarial nematodes endemic worldwide that infect dogs but are also zoonotic. Traveling dogs may serve as carriers of zoonotic parasites, including *Dirofilaria immitis,* introducing them to new regions. However, the risks of importation from endemic areas are unknown.

**Methods:**

To study seroprevalence in travelling dogs, we first established the signal-to-positive (S/P) cutoffs for the DiroCHEK^™^ ELISA assay (Zoetis) by spectrophotometry at three wavelengths (490, 620, or 650 nm). We also validated the assay for testing serum pools using known *D. immitis*-positive serum. We tested sera collected from 6699 dogs traveling from 34 countries between September 2022 and December 2023 using the spectrophotometric method. Samples were tested in serum pools initially, with pool sizes ranging from 5 to 15 samples based on reported prevalence. All pools were tested with and without heat treatment to disassociate immune complexes. Where sample numbers were insufficient to form a pool, samples were tested individually without heat treatment. Dogs in pools that tested positive were retested individually without heat treatment. Prevalence was calculated from pooled testing and individual testing results.

**Results:**

We tested 649 serum pools, of which 50 heat-treated pools and 11 non-heat-treated pools tested positive spectrophotometrically. Of these 61 positive pools, 7 tested positive in non-heat treated and heat-treated conditions, resulting in 54 unique pools. During re-testing of individual dogs from the positive pools, 31 pools contained dogs infected with *D. immitis*, while 23 pools had no positive individuals identified, indicating the presence of cross-reactive nematodes or antigen–antibody complexes. The prevalence of *D. immitis* and cross-reactive nematodes based on pooled testing was 0–5.32%. Due to the unavailability of specific serological tests for cross-reactive nematodes, they were not identified further. The prevalence of *D. immitis* based on individual testing was 0–11.1%, which was lower than the reported prevalence in their countries of origin.

**Conclusions:**

Our findings indicate that traveling dogs may carry *D. immitis* and cross-reactive nematodes, increasing the risk of introducing these parasites and/or novel parasite genetics to new areas. While pooled testing allows for screening populations rapidly, this methodology may underestimate true prevalence, particularly in low-prevalence regions.

**Graphical Abstract:**

**Figure:**
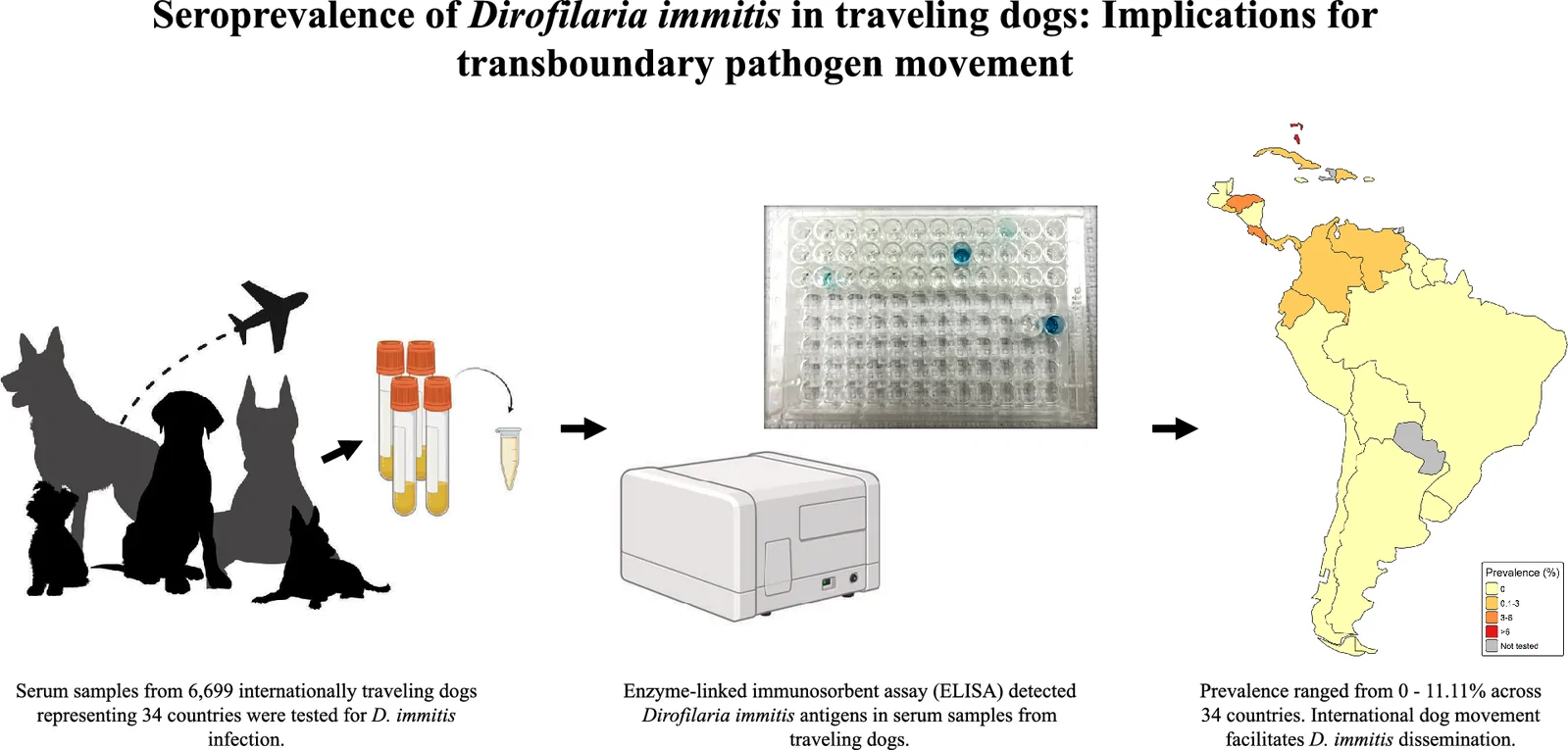

**Supplementary Information:**

The online version contains supplementary material available at https://doi.org/10.1186/s13071-026-07540-9.

## Background

The global movement of animals can contribute to the spread of zoonotic diseases and pose potential risks to both human and animal health. Diseases such as rabies, echinococcosis, leishmaniasis, toxoplasmosis, giardiasis, dirofilariasis, and vectors such as ticks have been previously spread by animal movement [1, 2]. Of these, the genus *Dirofilaria* represents mosquito-borne zoonotic agents that are less well-known to human public health professionals and the general public compared to mosquito-borne viral diseases. Previous studies have shown that both *Dirofilaria immitis* and *D. repens* can spread from endemic to non-endemic areas by dog movement [3–5]. *Dirofilaria immitis* causes heartworm disease in dogs and cats, and pulmonary dirofilariasis in humans [6]. Cases of human dirofilariasis caused by both *D. immitis* and *D. repens* are on the rise globally [7, 8].

In dogs, *D. immitis* infections are typically diagnosed using microfilariae and/or antigen tests that detect female uterine antigen[9]. In the United States, there are several U.S. Department of Agriculture (USDA)-approved, commercially available antigen-detection enzyme-linked immunosorbent assay (ELISA) kits, in which the positive results are based on the visual inspection of color change of an antibody-coated spot or well. However, subjectivity can be introduced by individual interpretation of visual color changes due to variabilities in ambient lighting, reader experience, or limitations in visual acuity, and can be overcome using spectrophotometry [10]. Additionally, while antigen tests have high specificity, sensitivity can vary considerably due to testing too early in infections prior to the development of adult female worms, low worm burdens, single-sex infections, and the formation of antigen–antibody complexes that interfere with antigen detection [9]. To dissociate immune complexes, heating serum to 100 °C for 10 min has been recommended by multiple researchers [9–14]; however, this process may increase the likelihood of cross-detection of other parasites like *Spirocerca lupi*, *Angiostrongylus vasorum*, and *D. repens* without the ability to differentiate them [15–18].

National and international veterinary authorities, including the Centers for Disease Control and Prevention (CDC), the USDA, the World Organization for Animal Health (WOAH), and national ministries of agriculture and public health have established guidelines for the movement of dogs to prevent the introduction of high-consequence zoonotic diseases, such as rabies, by requiring mandatory vaccination and testing. However, international dog movement also poses a risk for the transboundary spread of vector-borne and parasitic diseases. At present, import and export guidelines do not adequately address significant helminth diseases, such as dirofilariasis. The current process for determining a dog's health prior to import relies on obtaining a certificate from a licensed veterinarian in the country of origin, often within 24 h of departure. Many countries also require veterinary inspection at the port of entry, where signs of illness, regardless of prior certification, may result in denial of entry. However, regional differences in disease prevalence and access to diagnostic and treatment resources can influence the scope of health assessments. Globally, testing for *D. immitis* and other parasitic nematodes are not routine requirements for dog importation, with few exceptions such as South Africa [19], Iceland [20], and New Zealand [21].

*Dirofilaria immitis* is widespread throughout the world across temperate, subtropical and tropical regions, with higher levels of prevalence seen in tropical and subtropical climates [6, 22–26]. In the Asia-Pacific, *D. immitis* is present throughout South and Southeast Asia [27–32], East Asia [33], and Australia [34–37], though prevalence varies considerably between and within countries. In Europe, heartworm distribution shows marked regional variation. While the parasite is endemic across many temperate regions [8, 24, 26, 38, 39], prevalence rates are typically lower in northern areas. In contrast, Mediterranean countries including Italy, Spain, Greece, and Portugal represent significant endemic hotspots with substantially higher infection rates [38]. The parasite has also been documented in parts of the Middle East [26, 40], and North and sub-Saharan Africa [26, 40, 41], though data from these regions remain limited.

*Dirofilaria immitis* is prevalent throughout most of the Americas [42–44], including as far north as Canada [45, 46]. In the U.S., the parasite is endemic, with prevalence ranging between 0.1% and 8.1% depending on the region [47, 48]. However, pooled prevalence across both North and South America was considerably higher at 11.6% (CI 10.47–12.77%) in a meta-analysis [6]. In many Central and South American countries, the environmental conditions are highly conducive to mosquito vector survival [49], however *D. immitis* larval development within the mosquito is not well characterized. Additionally, access to heartworm prophylactics and the adulticidal treatment melarsomine remains limited in many Central and South American countries [22]. Melarsomine is the only Food and Drug Administration (FDA)-approved adulticidal treatment for *D. immitis* in the U.S. and is registered for use in only two other countries, Mexico and Argentina, in the Americas. The drug is banned in Colombia due to its arsenic content [22]. Thus, infected dogs may remain untreated and serve as reservoirs for continued transmission. Although surveillance data for *D. immitis* across Central and South America have historically been inconsistent, in recent years there have been regional increases in epidemiological monitoring [44]. Nonetheless, gaps remain in standardized data collection and create challenges when trying to assess the prevalence of *D. immitis* and cross-reactive nematodes.

The objective of this study was to determine whether dogs from 34 Central and South American countries were infected with *D. immitis* using a commercial well-based ELISA (DiroCHEK™, Zoetis Inc.) for antigen detection. We first aimed to validate the optimal wavelength and signal-to-positive (S/P) ratio cutoff for the DiroCHEK^™^ test kit as an alternative to the manufacturer-recommended visual interpretation [50] to help establish a binary classification system in which samples were designated as clearly positive or negative, avoiding indeterminate zones and reducing variability from subjective visual assessment [51]. We then evaluated the performance of a pooled sampling approach using known positive and negative canine serum. Finally, we pooled and tested canine serum samples using the optimized wavelength and S/P ratio cutoff to detect *D. immitis*-positive pools. We then retested all the individuals in positive pools to identify *D. immitis*-positive dogs.

## Methods

### Determination of the appropriate wavelength and diagnostic cutoff for ELISA interpretation

We determined the cutoff for the 490, 620, and 650 nm wavelength using a twofold serial dilution of the DiroCHEK^™^ Heartworm Antigen test kit’s positive control (Zoetis Inc., Kalamazoo, MI) from 1:4 to 1:4,096 dilutions (dilution factor range 0.25 to 2.44 × 10^–4^), tested in triplicate. We used 50 µL each of the positive and negative control solutions, measured using calibrated micropipettes, to ensure consistency of control wells across all runs in the study. We normalized optical density (OD) values by calculating the S/P ratio, dividing each sample’s OD value by that of the undiluted positive control sample in each run. We performed one-hot encoding based on the visualization of blue color as recommended by the manufacturer. We analyzed the normalized values using receiver operating characteristic (ROC) curves in R (version 4.3.2) using *plotROC* (version 2.3.1) and *pROC* (version 1.18.5) to evaluate the diagnostic accuracy of the assay. We identified the optimal cut-off threshold using Youden J statistic estimation using functions in the *pROC* library [52]. To estimate the analytical limit of detection of the DiroCHEK^™^ test kit visually as recommended by the manufacturer, we used the same positive control antigen dilution series described above. We used the final dilution at which blue color was still visibly detectable as the threshold for determining the lowest detectable antigen concentration for the assay.

### Validation of ELISA for pooled sampling

Three previously confirmed positive *D. immitis* serum samples from Georgia, USA, were used to validate a pooled sampling strategy. We diluted each sample at ratios of 1:5, 1:10, 1:15, 1:20, 1:50, and 1:100 (dilution factor range 0.01 to 0.5; undiluted serum was also tested as dilution factor 1) in U.S. Origin Canine Beagle Serum (Innovative Research, Novi, MI). From each dilution, 500 µL was aliquoted and heat-treated at 100 ℃ for 10 min, followed by centrifugation at 16,000 × g for 10 min. 50 µL of heat-treated and non-heat-treated serum were tested for *D. immitis* antigen using the DiroCHEK^™^ test kit. Wells were visually inspected for color change and absorbance measured on a spectrophotometer at wavelengths of 490, 620, and 650 nm. Dilution curves for visualization were plotted in R.

### Collection and composition of canine serum samples

Dog serum samples used for this study consisted of residual samples submitted to the Kansas State Veterinary Diagnostic Laboratory between September 2022 and December 2023 for pre-export testing. A convenience-based sampling strategy was used. The samples were distributed across 34 countries in Central and South America. All samples were anonymized with the only exception of information on the state/province and country of origin, which were retained. In total, we tested serum from 6699 dogs, with samples originating from each of the regions represented (Table 2). We assumed all dogs included in the study had access to some prior veterinary care to facilitate collection and submission of a serum sample.

### Pooled sampling

We determined pool sizes for each country and region by reviewing published prevalence estimates of *D. immitis* infections in dogs (Supplemental Table 1). When no data were available, older than 2010, or potentially underestimated (where neighboring countries had significantly higher reported prevalence), we referenced neighboring countries’ prevalence estimates. Using Epitools [53], pool sizes were calculated based on these prevalence estimates (Table 1). We capped pool sizes to a minimum of 5 and a maximum of 15 samples per pool. In total, twenty countries had enough available serum samples to allow for pooled testing, while samples from the remaining 14 countries were tested individually.

**Table 1:** Summary of pooled testing data and prevalence estimates by country

Country	Pooled serum testing							Individual serum testing			Total dogs represented (*n*)
	Prevalence used to calculate pool sizes	Serum pool size	Pools tested (*n*)	Dogs represented in pooled testing (*n*)	Pools positive (no heat treatment)	Pools positive (after heat treatment)	Calculated prevalence (pooled testing) % (CI), SD	Individuals in positive pools (*n* positive)	Additional individual samples tested (*n*)†	Calculated prevalence (individual testing) % (CI)	
Region: Central America											
Belize	*	5	2	10	0	1	0	0	2	0 (-, 18.4)	12
Costa Rica	10%*	15	15	225	1	8	4.95 (2.0–9.8); ± 1.75	14	12	5.91 (3.55–9.67)	237
El Salvador	*	5	67	335	0	5	1.54 (0.5–3.56); ± 0.68	2	2	0.59 (0.16–2.14)	337
Guatemala	30%	5	42	210	0	2	0.97 (0.12–3.46); ± 0.68	0	3	0 (-, 1.25)	213
Honduras	30%	5	46	230	3	11	5.32 (2.65–9.34); ± 1.57	8	4	3.42 (1.74–6.6)	234
Nicaragua	*	5	3	15	0	0	0	0	4	0 (-, 12.46)	19
Panama	*	15	8	120	0	3	3.1 (0.59–8.95); ± 1.77	3	9	2.33 (0.79–6.61)	129
Region: Greater Antilles											
Cuba	13%	12	54	648	0	4	0.64 (0.17–1.63); ± 0.32	2	11	0.30 (0.08–1.1)	659
Dominican Republic	18%	8	44	352	1	1	0.29 (0.01–1.59); ± 0.29	1	6	0.28 (0.05–1.57)	358
Puerto Rico	20%	8	2	16	0	0	0	0	3	0 (-, 12.46)	19
Region: South America											
Argentina	17%	9	6	54	0	0	0	0	8	0 (-, 4.18)	62
Bolivia	33%	5	17	85	1	0	1.2 (0.03–6.54); 1.2	0	783	0 (-, 3.02)	868
Brazil	2.2%*	15	10	150	0	1	0.70 (0.02–3.85); 0.7	0	6	0 (-, 1.7)	156
Chile	*	5	2	10	0	0	0	0	0	0 (-, 21.29)	10
Colombia	11%	14	150	2100	1	6	0.34 (0.14–0.70); ± 0.12	9	2	0.43 (0.23–0.81)	2102
Ecuador	15%	10	76	760	0	2	0.27 (0.03–0.96); ± 0.19	1	1	0.13 (0.02–0.74)	761
Peru	5%*	15	41	615	3	3	0.68 (0.18–1.74); ± 0.34	0	17	0 (-, 0.81)	632
Suriname	22%	5	5	25	0	0	0	0	1	0 (-, 9.43)	26
Uruguay	*	9	29	261	0	0	0	0	0	0 (-, 1.03)	261
Venezuela	15%	10	30	300	1	3	1.42 (0.38–3.6); ± 0.71	2	26	0.61 (0.17, 2.21)	326

*Prevalence data were either unavailable, outdated, or potentially underestimated; therefore, prevalence was inferred from surrounding countries with higher reported rates or estimated using a conservative pool size of 5 or a maximum pool size set to 15

^†^These were additional samples which were too few for pooling

Pool sizes were calculated in Epitools [53]. For countries with zero prevalence, the upper limit of the 90% confidence interval is reported; for countries with non-zero prevalence, 95% confidence intervals are provided

Serum samples from individuals were sorted by country and randomly allocated into pools. Each pool was a mixture of at least 100 µL of serum from each individual sample. These pools were each vortexed for 10 s, with 75 µL aliquoted and set aside as the non-heat-treated sample. We then heat-treated the remaining pooled sample at 100 ℃ for 10 min, followed by centrifugation at 16,000 × g for 10 min. We tested 50 µL of both heat-treated and non-heat-treated serum for *D. immitis* antigen using the DiroCHEK^™^ test kit. We visually inspected wells for color change as per the manufacturer’s recommendations and measured absorbance on a spectrophotometer at wavelengths of 490, 620, and 650 nm. We normalized OD readings by calculating the S/P ratio.

Individual samples in pools that tested positive for *D. immitis* (S/P ratio reading above the cutoff) were re-tested using only non-heat-treated serum with the DiroCHEK™ test kit and spectrophotometry.

### Analysis

We calculated apparent prevalence using the “Estimated true prevalence and predictive values from survey testing” tool in Epitools, applying the Wilson method [54]. We estimated pooled prevalence using the “Pooled prevalence for fixed pool size and perfect tests (method 2)” tool in Epitools [55]. We evaluated prevalence assuming perfect sensitivity and specificity, as claimed by the manufacturer, for infections with greater than three adult female *D. immitis* worms [56]. Visualizations were performed in R (version 4.3.2), using libraries *tidyverse* (version 2.0.0), *ggprism* (version 1.0.5), and *patchwork* (version 1.2.0).

## Results

### Determination of the appropriate wavelength and diagnostic cutoff for ELISA interpretation

**Fig. 1:**
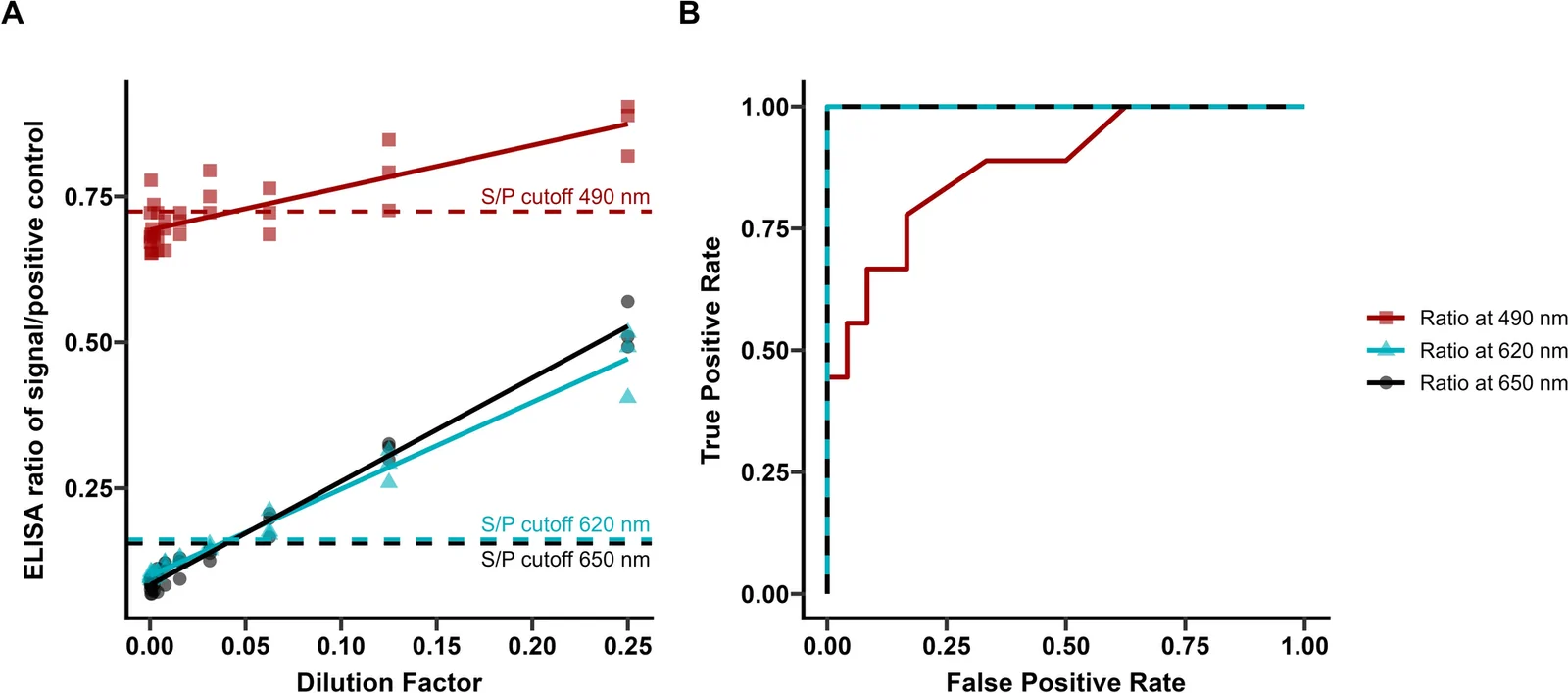
Determination of optimal spectrophotometric wavelength for the DiroCHEK™ test kit, an enzyme-linked immunosorbent assay (ELISA). **A** ELISA signal-to-positive control (S/P) ratios for twofold dilutions (1:4 to 1:4,096; dilution factor range 0.25 to 2.44 × 10–4) of the DiroCHEK™ test kit supplied positive control. Spectrophotometric data were collected at 490, 620, and 650 nm. Dotted lines represent S/P cutoffs at 490 (red), 620 (teal), and 650 (grey) nm. **B** Combined receiver operating characteristic (ROC) curves for 490 (red), 620 (teal), and 650 (grey) nm

To determine the optimal spectrophotometric wavelength for the DiroCHEK^™^ test kit, we conducted ROC analysis with S/P values at 490, 620, and 650 nm, which had previously been used in other studies [57–59] to select the wavelength that maximized sensitivity and specificity. For both the 620 nm and 650 nm, the area under the curve (AUC) was calculated as 1.00 (Fig. 1). The AUC for 490 nm was 0.877. We selected 650 nm for all subsequent analyses, consistent with manufacturer guidance prior to 2015 and the methods of Gruntmeir et al. [57], as well as other previously published methods [60]. The optimal cut-off threshold at 650 nm for classification as positive or negative using the Youden J statistic was determined to be 0.155, which provided a sensitivity of 1.00 and a specificity of 1.00. The cut-off thresholds for S/P values for 490 nm and 620 nm were 0.724 and 0.162, respectively. The limit of detection was determined to be 1:16 (dilution factor 0.0625) dilution of the positive control provided in the kit.

### Validation of pooled sampling

**Fig. 2:**
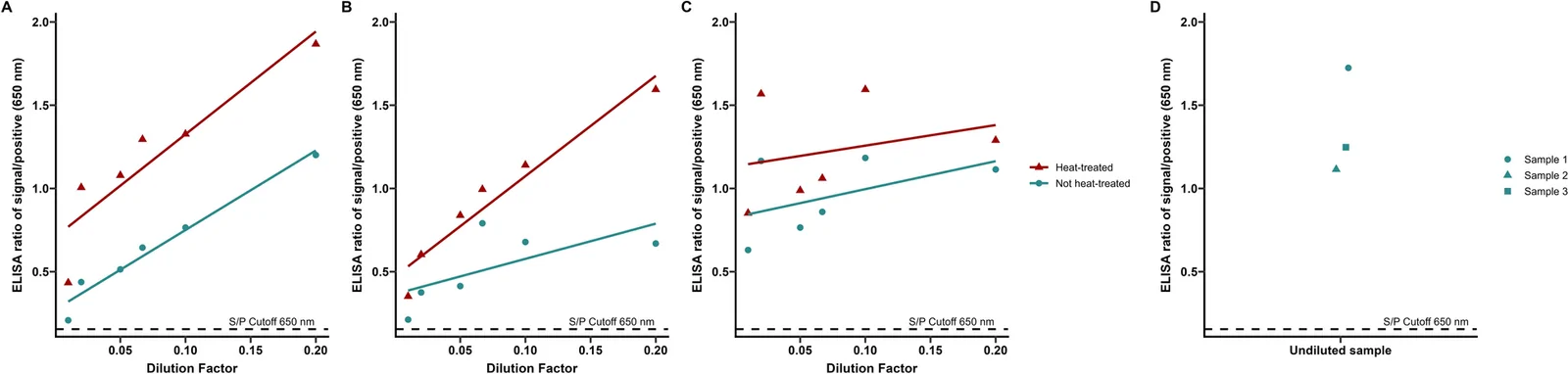
Validation of pooled sampling using serum from known *Dirofilaria immitis*-infected dogs. Enzyme-linked immunosorbent assay (ELISA) signal-to-positive control (S/P) ratios for serial dilutions of three confirmed positive canine serum samples with and without heat-treatment at 650 nm are shown for sample 1 (**A**), sample 2 (**B**), and sample 3 (**C**). S/P ratios for each of the three samples at a 1:1 (undiluted) concentration without heat-treatment are shown in **D**. The dotted line represents the S/P cutoff at 650 nm of 0.155

To determine if the DiroCHEK^™^ test kit could be used for pooled sampling, we performed a dilution series using three *D. immitis*-confirmed positive canine serum samples. For all dilutions, wells were visually positive (blue color change), and the S/P ratio values were above the cutoff threshold determined for the assay (Fig. 2).

### Pooled sampling and prevalence

**Fig. 3:**
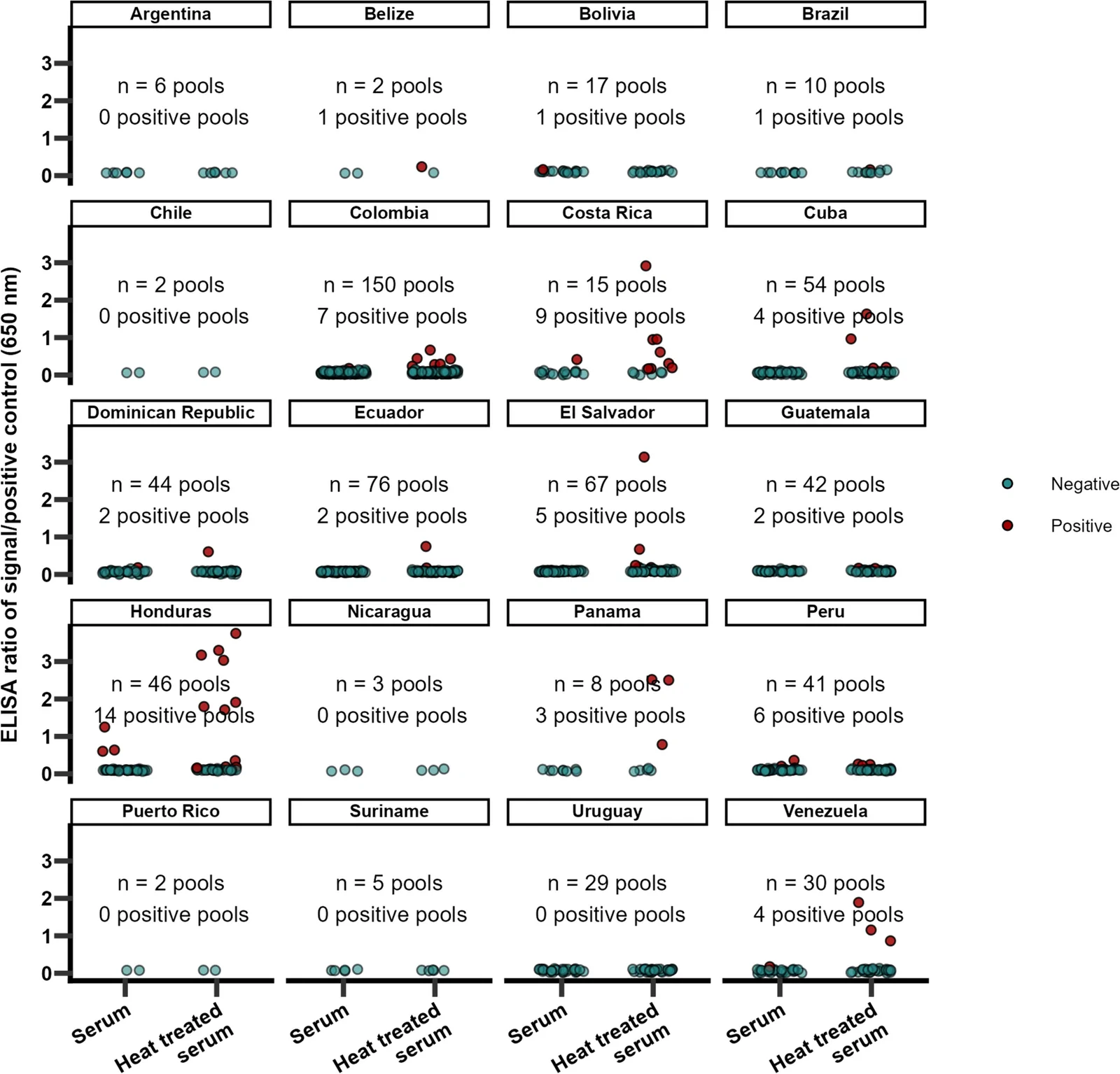
Pooled sample testing of canine sera from 20 Central and South American countries using spectrophotometry. Each dot represents a sample pool tested using the DiroCHEK^™^ test kit. Enzyme-linked immunosorbent assay (ELISA) results are expressed as the ratio of the sample signal-to-positive control (S/P). Red dots indicate pools that tested positive (S/P ≥ 0.155); teal dots indicate negative pools. Serum samples were tested with and without heat treatment

To screen traveling dogs from the various regions for *D. immitis* infection, we employed a pooled sampling strategy using the DiroCHEK™ test kit with the optimal threshold cutoff determined above for 650 nm (S/P ratio > 0.155). In total, 649 pools from 20 countries were included in the pooled sampling strategy. If a pool was negative, all individuals within the pool were considered negative. Additionally, 14 countries did not have enough samples to conduct pooled testing. Instead, all samples for these countries were tested individually with results reported either by country or grouped by region. As shown in Fig. 3 and Table 1, we detected positive pools in multiple countries across all sampled regions, including South America (6 countries), Central America (6 countries), and the Greater Antilles (2 countries).

**Fig. 4:**
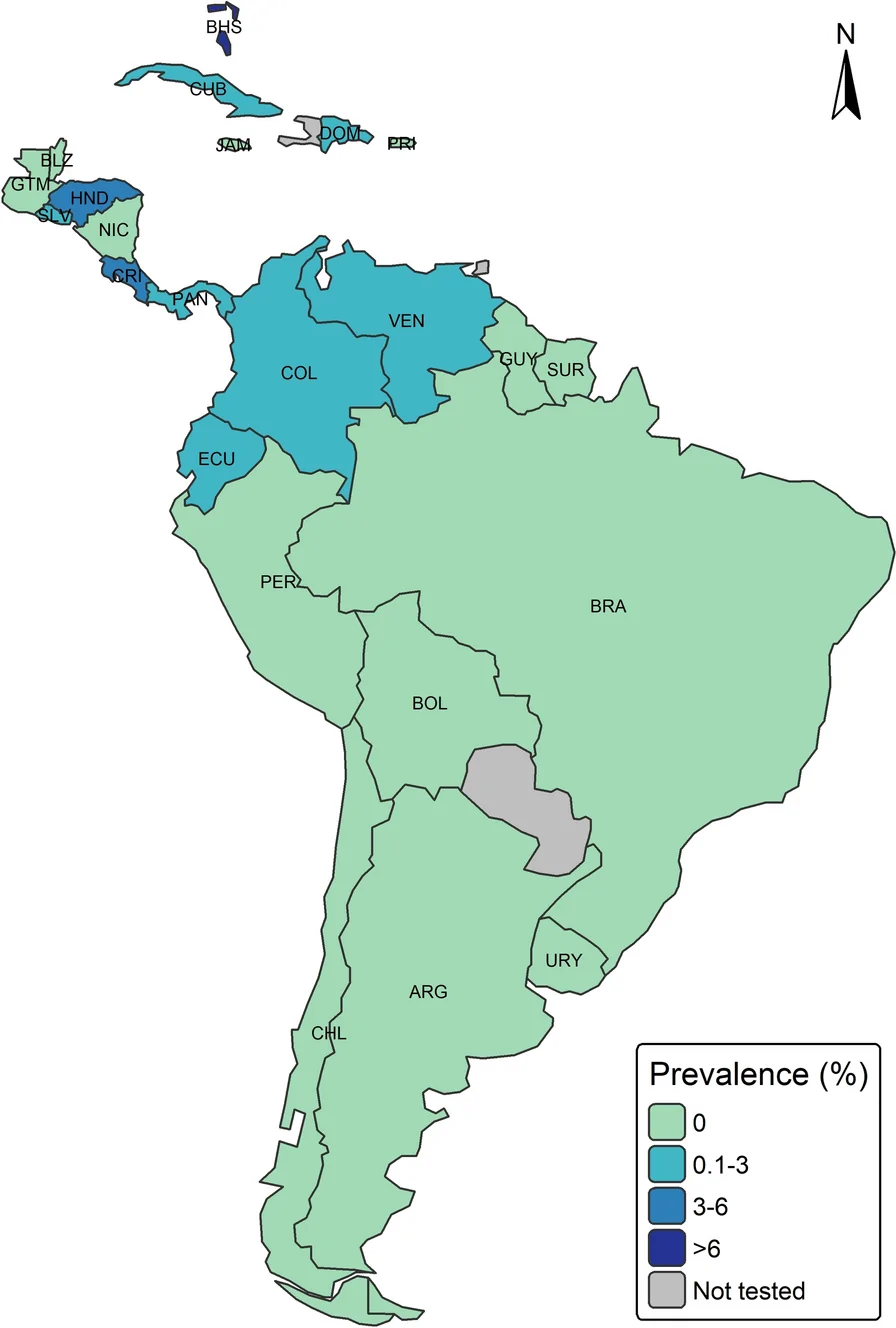
Prevalence map of *Dirofilaria immitis* infection in dogs traveling from Central and South American countries, based on samples tested in this study. Countries without available samples for testing are denoted in grey

To determine seroprevalence for *D. immitis* in traveling dogs from each country, we retested all individuals within positive pools using non-heat-treated serum to identify positive dogs. Apparent and pooled prevalence are shown in Fig. 4 and Tables 1 and 2. In contrast, despite previous reports of prevalence, no positive individuals were found in Argentina, Bolivia, Brazil, Nicaragua, Peru, or Puerto Rico. No prior reports were available for Chile or Uruguay, and no positive individuals were found in these countries. We found a total of 23 positive pools that had no individuals test positive when retested from Belize, Bolivia, Brazil, Colombia, Costa Rica, Cuba, Ecuador, El Salvador, Guatemala, Honduras, Peru, and Venezuela. Of the 14 countries tested individually, only the Lucayan Archipelago had positive individuals (2 of 18 samples), with an apparent prevalence of 11.1%.

**Table 2:** Summary of countries or regions not tested via the pooled testing strategy

Country	Number of individuals tested	Number of individuals positive	Calculated prevalence (%)	Lower CI (%)	Upper CI (%)
Guyana	1	0	0.0	–	65.5
Jamaica	9	0	0.0	–	23.11
Lesser Antilles	19	0	0.0	–	12.46
Lucayan Archipelago	18	2	11.11	3.1	32.8
Other Caribbean islands	12	0	0.0	–	18.4

For countries with zero prevalence, the upper limit of the 90% confidence interval is reported; for countries with non-zero prevalence, 95% confidence intervals are provided

## Discussion

There is a critical need to test traveling dogs for vector-borne parasitic infections, including *Dirofilaria* species, as the movement of infected undiagnosed dogs can contribute to the geographic spread of *D. immitis*. Even with routine testing of dogs, cases may be missed due to false-negative test results. False-negatives can result due to the parasite’s long prepatent period, during which circulating microfilariae and detectable antigen levels may be absent, as well as single-sex infections, low worm burdens, and immune complexes interference with antigen test sensitivity [9]. In this study, we aimed to investigate the seroprevalence of *D. immitis* in dogs moving between countries by evaluating a pooled sampling method for screening the population of traveling dogs. Our results indicate that a portion of dogs traveling internationally are seropositive for *Dirofilaria* spp. and specifically *D. immitis* (Fig. 3, Tables 1 and 2). Understanding the prevalence of *D. immitis* in traveling dogs is essential to mitigate the risk of introducing heartworm disease to non-endemic areas or novel genetics to endemic areas, and to inform effective prevention strategies.

The manufacturer’s instructions for the DiroCHEK^™^ test kit recommend evaluating test results visually by assessing a colorimetric change to blue after a 5-min incubation [50]. To improve precision and reproducibility, several studies have used spectrophotometric measurement of optical density (OD) values to quantify colorimetric changes more objectively at different wavelengths [12, 57–59]. One approach to differentiate positive from negative samples is to define a threshold for positivity as an OD value greater than or equal to that of the positive control. Samples with OD values below this threshold but greater than three standard deviations above the mean OD of negative controls are classified as “borderline”, creating a diagnostic gray zone [51]. However, in this approach, faint color changes resulting in lower OD values which do not approach the OD value of the positive control are misclassified. To avoid variations in OD values between runs and plates, we used signal-to-positive (S/P) values, an approach standard to ELISA-based spectrophotometry. To avoid the indeterminate zone, we used dilutions of the positive control provided in the kit to obtain S/P values to construct ROC curves and determine an optimal S/P cutoff. In our study, we have demonstrated that 650 nm is the optimal wavelength for use with the DiroCHEK^™^ test kit, having an area under the curve of 1, indicating 100% sensitivity and specificity (Fig. 1). We calculated the S/P cutoff to be 0.155 and used this derived threshold consistently across all pooled and individual sample results to standardize the test interpretation. This objective standardization enhances the reliability and comparability of results across ELISA plates and strengthens the validity of pooled surveillance for *D. immitis*. We recommend the establishment of cutoffs prior to experimental work in future spectrophotometric studies.

To screen for *D. immitis* infection in dogs, we evaluated a pooled sampling approach using the DiroCHEK^™^ test kit. This approach has shown success in similar pooled surveillance efforts for other infectious diseases such as malaria [61], COVID-19 [62], and HIV [63], as well as for blood bank safety screening efforts [64]. Pooled testing has been shown to be advantageous in low-prevalence settings, where it can improve testing efficiency without substantially compromising accuracy [65]. We used country-level estimates of *D. immitis* prevalence to guide our selection of pool sizes, which ranged from a minimum of 5 to a maximum of 15 samples per pool (Supplemental Table 1). The choice of pool size represents a cost–benefit tradeoff. Smaller pools are more sensitive, reducing the risk of missed infections and minimizing the burden of retesting. In contrast, larger pools are more cost-efficient, reducing the number of tests needed, but in areas of high disease prevalence, they increase the likelihood of requiring extensive follow-up testing when a pool tests positive. Our results indicate that the DiroCHEK^™^ test kit was able to detect a single known infected sample within a pool of up to 100 samples, both in non-heat-treated and heat-treated pooled sera (Fig. 2). These findings support the feasibility of pooled antigen testing as a scalable and resource-conscious surveillance strategy for *D. immitis*, especially in regions with limited diagnostic infrastructure.

Canine movement, whether transient or permanent, can influence the geographic distribution of *D. immitis* [66]. We detected *D. immitis*-positive pools and individuals in dog populations from Central and South America, and the Caribbean (Fig. 3, Tables 1 and 2). In the 20 countries where samples were tested using the pooled strategy, we found that apparent prevalence ranged from 0 to 5.9%. In this study, pool sizes were guided by previous prevalence data and constrained within a defined range of 5–15 individuals per pool. Even within this range, false-negative results remain possible in very low-prevalence settings. In cases where prevalence data are sparse or inconsistent, a pre-defined pooling strategy is recommended to ensure that results are reliable. In contrast, among the 14 countries where samples were tested individually, though with smaller sample sizes, prevalence ranged from 0 to 11.1%. Notably, the highest prevalence estimates we observed were in three Central American countries. Across all countries, our estimates were on average lower than previously reported values (Tables 1 and 2), which may reflect differences in our study population, as well as the inherent limitations of the pooling methodology used. This sampling may have resulted in an underestimation of true prevalence particularly in low-prevalence regions.

The detection of *D. immitis*-positive dogs in this study aligns with previous studies demonstrating that travel and relocation can facilitate the introduction of *D. immitis* into regions where transmission was previously not endemic or was limited [5, 67–69]. Natural disasters, like hurricane Katrina in 2005, have further exacerbated the spread through displacement of large populations of dogs from the Gulf Coast and southern states [69].

The global distribution and prevalence of *D. immitis* varies substantially by region [25, 37, 38, 40, 42, 44]. In the U.S., prevalence ranges from 1 to 12%, although highly endemic areas can reach up to 40% [42, 48]. In Canada, prevalence ranges from 0.2 to 3.9% [45, 46], while Mexico shows an average of 22.6%, with some areas as high as 64% [14]. Across the diverse climates and landscapes of Central and South America, prevalence is highly variable (Supplemental Table 1), ranging from 0.1 to 69% [42, 44], and our findings were consistent with this variability, though generally lower than previously reported values as discussed above. In Europe, Mediterranean countries demonstrate substantially higher rates, with prevalence ranging from 3.6% in Portugal to 6.5% in Spain nationally, though regional hotspots may exceed 40% in southern Italy and southeastern Romania [38]. Central and northern European countries typically show lower prevalence, with many cases attributed to imported infections, though endemic transmission is emerging in Germany, Austria, and Poland [38]. A recent meta-analysis estimated the combined prevalence of *Dirofilaria* spp. at 2.4% across North Africa and the Middle East [40]. In Africa and the Middle East, comprehensive surveillance data remain limited. Throughout Asia*, D. immitis* prevalence is highly variable within and between countries. Taiwan reports 57% in stray dogs and 30% in owned dogs on the main island [33, 70], India 1.5–18% depending on region [27, 32], Cambodia 15.8% [29], Malaysia 3.85–6.5%, Thailand 0 –48% across districts [70, 71], and 28% in Myanmar [72]. Australia reports pooled prevalence ranging from 2.1 to 18.7% across different climate zones [37]. These regional differences underscore the heterogeneous nature of heartworm distribution globally. Understanding regional baseline prevalence is essential for contextualizing surveillance findings and assessing the potential for parasite establishment in new areas. This is particularly relevant in the context of international dog movement, as demonstrated by our findings of *D. immitis*-positive dogs originating from multiple Central and South American countries and the Caribbean.

Furthermore, in recent years there has been an emergence of macrocyclic lactone-resistant *D. immitis* strains in the southern U.S. [73–79]. The recent detection of the first macrocyclic lactone-resistant case in Italy, traced to a dog imported from the U.S., demonstrates that international animal movement also poses a realistic risk for the global spread of drug-resistant strains [80]. Given the extensive global movement of animals, as demonstrated by the diversity of countries represented in our study, the need for efficient large-scale surveillance systems is critical to reduce the risk of parasite introduction.

Moreover, climate change is expanding the geographic range of competent vectors [81]. Several mosquito species are capable of serving as the intermediate host for *D. immitis* [82]. Our findings, in combination with these factors, highlight the need to proactively monitor, detect, and mitigate the spread of *D. immitis* across borders and into previously unaffected regions.

In addition to *D. immitis*, dogs can serve as carriers for other parasitic nematodes that may interfere with heartworm antigen diagnostics. These cross-reactive species include *Dirofilaria repens*, *Angiostrongylus vasorum*, and *Spirocerca lupi*, and are known to produce antigens that can cross-react with the DiroCHEK^™^ test kit, especially when heat-treatment is used to dissociate immune complexes [15–18]. However, the DiroCHEK^™^ test kit is unable to differentiate between these species. Moreover, *D. repens* is not currently endemic to the Americas, but it is a zoonotic species capable of causing human dirofilariasis. Multiple cases of *D. repens* have been documented in imported dogs in several European countries [4, 83, 84]. *A. vasorum* and *S. lupi*, while not zoonotic, have been reported in parts of Central and South America [85, 86], as well as the Caribbean [87]. *S. lupi* has also been reported in both wild and domestic canids in the southern U.S. [88]. Interestingly, we observed several heat-treated pooled samples that tested positive, yet none of the individual samples from those pools tested positive upon retesting with non-heat-treated serum. Given these discrepancies, it is plausible that some of these heat-treated positive pools could be due to cross-reactive parasitic nematodes. The absence of routine surveillance for these parasites makes it difficult to accurately assess the risk they pose to diagnostic specificity and limits our ability to confidently interpret unexpected antigen-positive results in pooled samples.

While our study has shown that the DiroCHEK^™^ test kit can be used for pooled sampling and that some dogs traveling from Central and South America are seropositive for *D. immitis*, several limitations are acknowledged. The canine population included in this study is not necessarily representative of the dog population in each country. Many of the dogs tested could have been personal pets traveling internationally with their owners, a population that typically has consistent access to veterinary care, including prophylactic heartworm treatments. In contrast, a proportion of these could have been dogs relocated for adoption purposes that may have unknown or inconsistent medical histories. Furthermore, it is important to note that dogs may not have originated from the exact geographic area in which their blood samples were collected and past exposures in other regions cannot be discounted. Although it is assumed for this study that dogs reside in the country from which their blood sample was submitted, the precise location where a dog may have acquired a *D. immitis* infection is often unknown. Additionally, while the DiroCHEK™ test kit demonstrates high sensitivity and specificity, particularly for patent infections with three or more mature adult female worms, pooling may dilute antigen concentrations, increasing the likelihood of false-negative results. Despite these limitations, all positive pools with individual positives identified in our study are likely to reflect true infections, underscoring the utility of this method for initial surveillance. Future work should refine pool sizes relative to true local prevalence and explore methods to enhance sensitivity in low-burden infections.

## Conclusions

The findings of this study demonstrate that international canine movement represents a pathway for the introduction and spread of *D. immitis* and cross-reactive nematode parasites into new geographic regions, with implications for both parasite establishment and the dissemination of novel parasite genetics. These results underscore the importance of ongoing surveillance for *Dirofilaria* spp. and other vector-borne infections, particularly in populations with high mobility, to better inform heartworm prevention strategies and global importation regulations. Additionally, incorporating parasite screening into importation testing policies could serve as a critical tool for monitoring and mitigating zoonotic risks associated with global animal movement.

## Supplementary Information

Supplementary Material 1.

## Data Availability

Datasets from this study are available in the Zenodo repository, https://doi.org/10.5281/zenodo.17437431. The repository includes anonymized spectrophotometric values for pooled samples tested by DiroCHEK ELISA and anonymized individual‑level results for dogs from positive pools. All sample identifiers have been anonymized, and no personally identifiable or individually identifiable animal information is included.

## References

[1] Fooks AR, Johnson N. Jet set pets: examining the zoonosis risk in animal import and travel across the European Union. Vet Med. 2015;6:17–25. 10.2147/vmrr.S62059. PMC6067792 30101093

[2] Tiffin HS, Rajotte EG, Sakamoto JM, Machtinger ET. Tick control in a connected world: challenges, solutions, and public policy from a United States border perspective. Trop Med Infect Dis. 2022. 10.3390/tropicalmed7110388. 36422939 PMC9695313

[3] Fuehrer HP, Auer H, Leschnik M, Silbermayr K, Duscher G, Joachim A. *Dirofilaria* in humans, dogs, and vectors in Austria (1978-2014)-from imported pathogens to the endemicity of *Dirofilaria repens*. PLoS Negl Trop Dis. 2016;10:e0004547. 10.1371/journal.pntd.0004547. 27196049 PMC4873239

[4] Pantchev N, Etzold M, Daugschies A, Dyachenko V. Diagnosis of imported canine filarial infections in Germany 2008–2010. Parasitol Res. 2011;109:61–76. 10.1007/s00436-011-2403-7. 21739376

[5] Sabūnas V, Radzijevskaja J, Sakalauskas P, Paulauskas A. First report of heartworm (*Dirofilaria Immitis*) infection in an imported dog in Lithuania. Helminthologia. 2019;56:57–61. 10.2478/helm-2018-0036. 31662673 PMC6662024

[6] Anvari D, Narouei E, Daryani A, Sarvi S, Moosazadeh M, Ziaei Hezarjaribi H, et al. The global status of *Dirofilaria immitis* in dogs: a systematic review and meta-analysis based on published articles. Res Vet Sci. 2020;131:104–16. 10.1016/j.rvsc.2020.04.002. 32330696

[7] Simón F, Diosdado A, Siles-Lucas M, Kartashev V, González-Miguel J. Human dirofilariosis in the 21st century: a scoping review of clinical cases reported in the literature. Transbound Emerg Dis. 2022;69:2424–39. 10.1111/tbed.14210. 34197050

[8] Hattendorf C, Lühken R. Vectors, host range, and spatial distribution of *Dirofilaria immitis* and *D. repens* in Europe: a systematic review. Infect Dis Poverty. 2025;14:58. 10.1186/s40249-025-01328-2. 40604914 PMC12217203

[9] American Heartworm Society. Canine guidelines for the prevention, diagnosis, and management of heartworm (*Dirofilaria immitis*) infection in dogs. 2024.

[10] Velasquez L, Blagburn BL, Duncan-Decoq R, Johnson EM, Allen KE, Meinkoth J, et al. Increased prevalence of *Dirofilaria immitis* antigen in canine samples after heat treatment. Vet Parasitol. 2014;206:67–70. 10.1016/j.vetpar.2014.03.021. 24785291

[11] DiGangi BA, Dworkin C, Stull JW, O’Quin J, Elser M, Marsh AE, et al. Impact of heat treatment on *Dirofilaria immitis* antigen detection in shelter dogs. Parasit Vectors. 2017;10:483. 10.1186/s13071-017-2443-7. 29143645 PMC5688474

[12] Gruntmeir JM, Long MT, Blagburn BL, Walden HS. Canine heartworm and heat treatment: an evaluation using a well based enzyme-linked immunosorbent assay (ELISA) and canine sera with confirmed heartworm infection status. Vet Parasitol. 2020;283:109169. 10.1016/j.vetpar.2020.109169. 32593059

[13] Little S, Saleh M, Wohltjen M, Nagamori Y. Prime detection of *Dirofilaria immitis*: understanding the influence of blocked antigen on heartworm test performance. Parasit Vectors. 2018;11:186. 10.1186/s13071-018-2736-5. 29554955 PMC5859648

[14] Hay-Parker S, Rodriguez-Vivas RI, Tobias E, Beugnet F, Montes N, García E, et al. Prevalence and risk factors of *Dirofilaria immitis* infection in dogs from Mexico, including serum pre-heat treatment for the dissociation of immune complexes. Curr Res Parasitol Vector-Borne Dis. 2025;8:100289. 10.1016/j.crpvbd.2025.100289. 40689038 PMC12275470

[15] Aroch I, Rojas A, Slon P, Lavy E, Segev G, Baneth G. Serological cross-reactivity of three commercial in-house immunoassays for detection of *Dirofilaria immitis* antigens with *Spirocerca lupi* in dogs with benign esophageal spirocercosis. Vet Parasitol. 2015;211:303–5. 10.1016/j.vetpar.2015.06.010. 26116456

[16] Schnyder M, Deplazes P. Cross-reactions of sera from dogs infected with *Angiostrongylus vasorum* in commercially available *Dirofilaria immitis* test kits. Parasit Vectors. 2012;5:258. 10.1186/1756-3305-5-258. 23148786 PMC3503614

[17] Sobotyk C, Savadelis MD, Verocai GG. Detection and cross-reaction of *Dirofilaria repens* using a commercial heartworm antigen test kit. Vet Parasitol. 2021;289:109302. 10.1016/j.vetpar.2020.109302. 33352522

[18] Venco L, Manzocchi S, Genchi M, Kramer LH. Heat treatment and false-positive heartworm antigen testing in ex vivo parasites and dogs naturally infected by *Dirofilaria repens* and *Angiostrongylus vasorum*. Parasit Vectors. 2017;10:476. 10.1186/s13071-017-2444-6. 29143662 PMC5688472

[19] Department of Agriculture LRaRD. Information document for import of dogs and cats into the Republic of South Africa. 2022.

[20] P. Willeberg. Consulting: risk assessment of import of dogs and cats to Iceland. 2019.

[21] Ministry for Primary Industries: Bringing your Dog to New Zealand. 2019.

[22] Dantas-Torres F, Ketzis J, Pérez Tort G, Mihalca AD, Baneth G, Otranto D, et al. Heartworm adulticide treatment: a tropical perspective. Parasit Vectors. 2023;16:148. 10.1186/s13071-023-05690-8. 37106364 PMC10141906

[23] Noack S, Harrington J, Carithers DS, Kaminsky R, Selzer PM. Heartworm disease - Overview, intervention, and industry perspective. Int J Parasitol Drugs Drug Resist. 2021;16:65–89. 10.1016/j.ijpddr.2021.03.004. 34030109 PMC8163879

[24] Simón F, Siles-Lucas M, Morchón R, González-Miguel J, Mellado I, Carretón E, et al. Human and Animal Dirofilariasis: the Emergence of a Zoonotic Mosaic. Clin Microbiol Rev. 2012;25:507–44. 10.1128/cmr.00012-12. 22763636 PMC3416488

[25] Vinnie-Siow WY, Tan TK, Sam SS, Teoh BT, Lim YA-L, Low VL. Global prevalence, diagnostics and epidemiological trends of canine filariosis and associated zoonotic infections (2021–2025): a review. Vet Res Commun. 2026;50:74. 10.1007/s11259-025-10987-3. 41400797

[26] Genchi C, Kramer LH. The prevalence of *Dirofilaria immitis* and *D. repens* in the Old World. Vet Parasitol. 2020;280:108995. 10.1016/j.vetpar.2019.108995. 32155518

[27] Borthakur SK, Deka DK, Islam S, Sarma DK, Sarmah PC. Prevalence and molecular epidemiological data on *Dirofilaria immitis* in dogs from Northeastern States of India. Sci World J. 2015;2015:265385. 10.1155/2015/265385. PMC4320797 25685835

[28] Fuehrer H-P, Treiber M, Silbermayr K, Baumann TA, Swoboda P, Joachim A, et al. Indigenous *Dirofilaria immitis* in Bangladesh. Parasitol Res. 2013;112:2393–5. 10.1007/s00436-013-3311-9. 23358737

[29] Inpankaew T, Hii SF, Chimnoi W, Traub RJ. Canine vector-borne pathogens in semi-domesticated dogs residing in northern Cambodia. Parasit Vectors. 2016;9:253. 10.1186/s13071-016-1552-z. 27161452 PMC4862146

[30] Kaikuntod M, Thongkorn K, Tiwananthagorn S, Boonyapakorn C. Filarial worms in dogs in Southeast Asia. Vet Integr Sci. 2018;16:1–17.

[31] Kunathasan Chelliah M, Šlapeta J. The prevalence and trends of canine heartworm (*Dirofilaria immitis*) in Kuala Lumpur, Malaysia (1970–2018). Vet Parasitol Reg Stud Rep. 2019;16:100272. 10.1016/j.vprsr.2019.100272. 31027591

[32] Megat Abd Rani PA, Irwin PJ, Gatne M, Coleman GT, McInnes LM, Traub RJ. A survey of canine filarial diseases of veterinary and public health significance in India. Parasit Vectors. 2010;3:30. 10.1186/1756-3305-3-30. 20377864 PMC2857864

[33] Wu C-C, Fan P-C. Prevalence of canine dirofilariasis in Taiwan. J Helminthol. 2003;77:83–8. 10.1079/JOH2002150. 12590670

[34] Panetta JL, Calvani NED, Orr B, Nicoletti AG, Ward MP, Šlapeta J. Multiple diagnostic tests demonstrate an increased risk of canine heartworm disease in northern Queensland, Australia. Parasit Vectors. 2021;14:393. 10.1186/s13071-021-04896-y. 34372886 PMC8351338

[35] Nguyen C, Koh WL, Casteriano A, Beijerink N, Godfrey C, Brown G, et al. Mosquito-borne heartworm *Dirofilaria immitis* in dogs from Australia. Parasit Vectors. 2016;9:535. 10.1186/s13071-016-1821-x. 27717406 PMC5055658

[36] Constantinoiu C, Croton C, Paterson MBA, Knott L, Henning J, Mallyon J, et al. Prevalence of canine heartworm infection in Queensland, Australia: comparison of diagnostic methods and investigation of factors associated with reduction in antigen detection. Parasit Vectors. 2023;16:63. 10.1186/s13071-022-05633-9. 36765417 PMC9921331

[37] Atkinson PJ, O’Handley R, Nielsen T, Caraguel CGB. Heterogeneous distribution of the reported prevalence of *Dirofilaria immitis* infections in Australian canids – a systematic review and meta-analysis. Prev Vet Med. 2025;237:106438. 10.1016/j.prevetmed.2025.106438. 39862448

[38] Morchón R, Montoya-Alonso JA, Rodríguez-Escolar I, Carretón E. What has happened to heartworm disease in Europe in the last 10 years? Pathogens. 2022;11:1042. 10.3390/pathogens11091042. 36145474 PMC9503846

[39] Széll Z, Bacsadi Á, Szeredi L, Nemes C, Fézer B, Bakcsa E, et al. Rapid spread and emergence of heartworm resulting from climate and climate-driven ecological changes in Hungary. Vet Parasitol. 2020;280:109067. 10.1016/j.vetpar.2020.109067. 32145530

[40] Izenour K, Salib F, Eckert J, Jesudoss Chelladurai JRJ, Starkey L, Blagburn B, et al. A meta-analysis on *Dirofilaria immitis* and *Dirofilaria repens* in countries of North Africa and the Middle East. Parasitology. 2025;152:347–65. 10.1017/S003118202500037X. 40167419 PMC12186096

[41] McCall JW, Genchi C, Kramer LH, Guerrero J, Venco L. Chapter 4 heartworm disease in animals and humans. In: Advances in parasitology, vol. 66. Academic Press; 2008. p. 193–285. 10.1016/S0065-308X(08)00204-2 18486691

[42] Dantas-Torres F, Otranto D. Overview on *Dirofilaria immitis* in the Americas, with notes on other filarial worms infecting dogs. Vet Parasitol. 2020;282:109113. 10.1016/j.vetpar.2020.109113. 32464570

[43] Labarthe N, Guerrero J. Epidemiology of heartworm: what is happening in South America and Mexico? Vet Parasitol. 2005;133:149–56. 10.1016/j.vetpar.2005.04.006. 16198820

[44] Maggi RG, Krämer F. A review on the occurrence of companion vector-borne diseases in pet animals in Latin America. Parasit Vectors. 2019;12:145. 10.1186/s13071-019-3407-x. 30917860 PMC6438007

[45] Jacobson LS, Ward KA, Lacaden AB, Hornak TA. Prevalence of heartworm in relocated, local and outreach clinic dogs: a Canadian sheltering perspective. Vet Parasitol. 2020;283:109081. 10.1016/j.vetpar.2020.109081. 32521391

[46] Evason M, Stull JW, Pearl DL, Peregrine AS, Jardine C, Buch JS, et al. Prevalence of *Borrelia burgdorferi*, *Anaplasma* spp., *Ehrlichia* spp. and *Dirofilaria immitis* in Canadian dogs, 2008 to 2015: a repeat cross-sectional study. Parasit Vectors. 2008;12:64. 10.1186/s13071-019-3299-9. PMC6350403 30691522

[47] Little S, Braff J, Place J, Buch J, Dewage BG, Knupp A, et al. Canine infection with *Dirofilaria immitis*, *Borrelia burgdorferi, Anaplasma* spp., and *Ehrlichia* spp. in the United States, 2013-2019. Parasit Vectors. 2021;14:10. 10.1186/s13071-020-04514-3. 33407758 PMC7789229

[48] Smith R, Murillo DFB, Chenoweth K, Barua S, Kelly PJ, Starkey L, et al. Nationwide molecular survey of *Dirofilaria immitis* and *Dirofilaria repens* in companion dogs and cats, United States of America. Parasit Vectors. 2022;15:367. 10.1186/s13071-022-05459-5. 36229848 PMC9559157

[49] Riahi SM, Yusuf MA, Azari-Hamidian S, Solgi R. Prevalence of *Dirofilaria immitis* in mosquitoes (*Diptera)* - systematic review and meta-analysis. J Nematol. 2021;53 :e2021-12. 10.21307/jofnem-2021-012. 33860239 PMC8039976

[50] Zoetis Inc.: DiroCHEK™ Heartworm Antigen Test Kit Package Insert. Kalamazoo, MI: Zoetis.

[51] Starkey LA, Bowles JV, Payton ME, Blagburn BL. Comparative evaluation of commercially available point-of-care heartworm antigen tests using well-characterized canine plasma samples. Parasit Vectors. 2017;10:475. 10.1186/s13071-017-2447-3. 29143666 PMC5688447

[52] Robin X, Turck N, Hainard A, Tiberti N, Lisacek F, Sanchez J-C, et al. pROC: an open-source package for R and S+ to analyze and compare ROC curves. BMC Bioinformatics. 2011;12:77. 10.1186/1471-2105-12-77. 21414208 PMC3068975

[53] Sergeant E. Epitools epidemiological calculators. Ausvet Fremantle: Australia; 2018.

[54] Brown LD, Cai TT, DasGupta A. Interval estimation for a binomial proportion. Stat Sci. 2001;16:101–33. 10.1214/ss/1009213286.

[55] Cowling DW, Gardner IA, Johnson WO. Comparison of methods for estimation of individual-level prevalence based on pooled samples. Prev Vet Med. 1999;39:211–25. 10.1016/s0167-5877(98)00131-7. 10327439

[56] McCall JW, Supakorndej N, Donoghue AR, Turnbull RK, Radecki SV. Evaluation of the performance of canine heartworm antigen test kits licensed for use by veterinarians and canine heartworm antigen tests conducted by diagnostic laboratories. Recent Advances in Heartworm Disease: Symposium 2001. Batavia, IL: American Heartworm Society. 2001:97–104.

[57] Gruntmeir JM, Abbott JR, Kima PE, Long MT, Blagburn BL, Walden HS. Increasing temperature denatures canine IgG reducing its ability to inhibit heartworm antigen detection. Parasit Vectors. 2023;16:152. 10.1186/s13071-023-05739-8. 37106356 PMC10142223

[58] Perles L, Carbonara M, Mendoza-Roldan JA, Venco L, Gabrielli S, Otranto D. An indirect ELISA for the detection of antibodies against *Dirofilaria* spp. in cats. Parasit Vectors. 2025;18:16. 10.1186/s13071-024-06657-z. 39828739 PMC11744980

[59] Savadelis MD, Roveto JL, Ohmes CM, Hostetler JA, Settje TL, Dzimianski MT, et al. Evaluation of heat-treating heartworm-positive canine serum samples during treatment with advantage multi^®^ for dogs and doxycycline. Parasit Vectors. 2018;11:98. 10.1186/s13071-018-2685-z. 29458396 PMC5819156

[60] Drake J, Gruntmeir J, Merritt H, Allen L, Little SE. False negative antigen tests in dogs infected with heartworm and placed on macrocyclic lactone preventives. Parasit Vectors. 2015;8:68. 10.1186/s13071-015-0698-4. 25648086 PMC4336501

[61] Chang M, Johnston S, Seilie AM, Hergott D, Murphy SC. Application of dried blood spot sample pooling strategies for *Plasmodium* 18S rRNA biomarker testing to facilitate identification of infected persons in large-scale epidemiological studies. Malar J. 2021;20:391. 10.1186/s12936-021-03907-8. 34620192 PMC8499573

[62] Abdalhamid B, Bilder CR, McCutchen EL, Hinrichs SH, Koepsell SA, Iwen PC. Assessment of specimen pooling to conserve SARS CoV-2 testing resources. Am J Clin Pathol. 2020;153:715–8. 10.1093/ajcp/aqaa064. 32304208 PMC7188150

[63] van Schalkwyk C, Maritz J, van Zyl GU, Preiser W, Welte A. Pooled PCR testing of dried blood spots for infant HIV diagnosis is cost efficient and accurate. BMC Infect Dis. 2019;19:136. 10.1186/s12879-019-3767-z. 30744605 PMC6371519

[64] Bilder CR, Tebbs JM. Pooled-testing procedures for screening high volume clinical specimens in heterogeneous populations. Stat Med. 2012;31:3261–8. 10.1002/sim.5334. 22415972 PMC3500568

[65] Tu XM, Litvak E, Pagano M. Screening tests: can we get more by doing less? Stat Med. 1994;13:1905–19. 10.1002/sim.4780131904. 7846399

[66] Wright I, Jongejan F, Marcondes M, Peregrine A, Baneth G, Bourdeau P, et al. Parasites and vector-borne diseases disseminated by rehomed dogs. Parasit Vectors. 2020;13:546. 10.1186/s13071-020-04407-5. 33168100 PMC7653694

[67] Alvarez Rojas CA, Cancino-Faure B, Lillo P, Fernández ML, González AP, Ramírez AF. *Dirofilaria immitis* in dog imported from Venezuela to Chile. Emerg Infect Dis. 2023;29:439–41. 10.3201/eid2902.221427. 36692864 PMC9881789

[68] Drake J, Parrish RS. Dog importation and changes in heartworm prevalence in Colorado 2013–2017. Parasit Vectors. 2019;12:207. 10.1186/s13071-019-3473-0. 31060572 PMC6501459

[69] Levy JK, Lappin MR, Glaser AL, Birkenheuer AJ, Anderson TC, Edinboro CH. Prevalence of infectious diseases in cats and dogs rescued following Hurricane Katrina. J Am Vet Med Assoc. 2011;238:311–7. 10.2460/javma.238.3.311. 21281213

[70] Kamyingkird K, Junsiri W, Chimnoi W, Kengradomkij C, Saengow S, Sangchuto K, et al. Prevalence and risk factors associated with *Dirofilaria immitis* infection in dogs and cats in Songkhla and Satun provinces, Thailand. Agric Nat Res. 2017;51:299–302. 10.1016/j.anres.2017.05.003.

[71] Jitsamai W, Piromkij P, Kamkong P, Chungpivat S, Taweethavonsawat P. Seasonal distribution and environmental parameters associated with *Brugia pahangi* and *Dirofilaria immitis* in naturally infected dogs in Bangkok and vicinity, Thailand. Sci Rep. 2021;11:4594. 10.1038/s41598-021-84215-8. 33633276 PMC7907406

[72] Bawm S, Khaing Y, Chel HM, Hmoon MM, Win SY, Bo M, et al. Molecular detection of *Dirofilaria immitis* and its *Wolbachia* endosymbionts in dogs from Myanmar. Curr Res Parasitol Vector-Borne Dis. 2023;4:100148. 10.1016/j.crpvbd.2023.100148. 38021190 PMC10665652

[73] Bourguinat C, Lee ACY, Lizundia R, Blagburn BL, Liotta JL, Kraus MS, et al. Macrocyclic lactone resistance in *Dirofilaria immitis*: failure of heartworm preventives and investigation of genetic markers for resistance. Vet Parasitol. 2015;210:167–78. 10.1016/j.vetpar.2015.04.002. 25936435

[74] Pulaski CN, Malone JB, Bourguinat C, Prichard R, Geary T, Ward D, et al. Establishment of macrocyclic lactone resistant *Dirofilaria immitis* isolates in experimentally infected laboratory dogs. Parasit Vectors. 2014;7:494. 10.1186/s13071-014-0494-6. 25376278 PMC4228187

[75] Curry E, Tack D, Rodriguez J, Brehm-Lowe D, Letherer J, Lineberry M, et al. Surveillance of single nucleotide polymorphisms correlated to macrocyclic lactone resistance in *Dirofilaria immitis* from client-owned dogs across the United States. Int J Parasitol Drugs Drug Resist. 2025;29:100604. 10.1016/j.ijpddr.2025.100604. 40779992 PMC12355919

[76] Curry E, Traversa D, Carretón E, Kramer L, Sager H, Young L, et al. *Dirofilaria immitis:* genotyping randomly selected European clinical samples and USA laboratory isolates with molecular markers associated with macrocyclic lactone susceptibility and resistance. Pathogens. 2022;11(8):934. 10.3390/pathogens11080934. 36015054 PMC9415351

[77] Fisher PT, Keller K, Prichard RK. Investigating *Dirofilaria immitis* isolates infecting domestic canines and their susceptibility/resistance patterns to macrocyclic lactones in the northern region of the Mississippi Delta area (southeast Missouri). Vet Parasitol. 2024;329:110199. 10.1016/j.vetpar.2024.110199. 38781830

[78] Ballesteros C, Pulaski CN, Bourguinat C, Keller K, Prichard RK, Geary TG. Clinical validation of molecular markers of macrocyclic lactone resistance in *Dirofilaria immitis*. Int J Parasitol Drugs Drug Resist. 2018;8:596–606. 10.1016/j.ijpddr.2018.06.006. 30031685 PMC6288007

[79] Diakou A, Prichard RK. Concern for *Dirofilaria immitis* and macrocyclic lactone loss of efficacy: current situation in the USA and Europe, and future scenarios. Pathogens. 2021;10:1323. 34684273 10.3390/pathogens10101323 PMC8541013

[80] Traversa D, Diakou A, Colombo M, Kumar S, Long T, Chaintoutis SC, et al. First case of macrocyclic lactone-resistant *Dirofilaria immitis* in Europe–cause for concern. Int J Parasitol Drugs Drug Resist. 2024;25:100549. 10.1016/j.ijpddr.2024.100549. 38795510 PMC11153229

[81] Wang D, Bowman DD, Brown HE, Harrington LC, Kaufman PE, McKay T, et al. Factors influencing U.S. canine heartworm (*Dirofilaria immitis*) prevalence. Parasit Vectors. 2014;7:264. 10.1186/1756-3305-7-264. 24906567 PMC4101712

[82] Bowman DD, Atkins CE. Heartworm biology, treatment, and control. Vet Clin Small Animal Pract. 2009;39:1127–58. 10.1016/j.cvsm.2009.06.003. 19932367

[83] Agapito D, Aziz NA, Wang T, Morgan E, Wright I. Subconjunctival *Dirofilaria repens* infection in a dog resident in the UK. J Small Anim Pract. 2018;59:50–2. 10.1111/jsap.12795. 29205353

[84] Wright I. Case report: *Dirofilaria repens* in a canine castrate incision. Companion Animal. 2017;22:316–8. 10.12968/coan.2017.22.6.316.

[85] De Aguiar I, García R, Madriz D, Alfaro-Alarcón A, Montenegro VM, Aizenberg I, et al. Esophageal spirocercosis with pulmonary egg deposition and secondary hypertrophic osteopathy in a dog from Costa Rica. Vet Parasitol Reg Stud Rep. 2021;23:100510. 10.1016/j.vprsr.2020.100510. 33678365

[86] Penagos-Tabares F, Lange MK, Chaparro-Gutiérrez JJ, Taubert A, Hermosilla C. *Angiostrongylus vasorum* and *Aelurostrongylus abstrusus*: neglected and underestimated parasites in South America. Parasit Vectors. 2018;11:208. 10.1186/s13071-018-2765-0. 29587811 PMC5870519

[87] Chikweto A, Bhaiyat M, Tiwari K, De Allie C, Sharma R. Spirocercosis in owned and stray dogs in Grenada. Vet Parasitol. 2012;190:613–6. 10.1016/j.vetpar.2012.07.006. 22841904

[88] Rojas A, Dvir E, Baneth G. Insights on *Spirocerca lupi*, the carcinogenic dog nematode. Trends Parasitol. 2020;36:52–63. 10.1016/j.pt.2019.10.004. 31734099

