# Supplementary material for: Seroprevalence of Dirofilaria immitis in traveling dogs: implications for transboundary pathogen movement
Source: Parasit Vectors. 2026 Jul 2;19:402. doi: 10.1186/s13071-026-07540-9 (PMC13595801; doi:10.1186/s13071-026-07540-9)
Supplement: Supplementary file 1 [file 13071_2026_7540_MOESM1_ESM.docx]

Appendix 1. Reported prevalence range of *Dirofilaria immitis* infection in dogs in Central and South American countries.

| **Country** | **Reported Prevalence** | **Reference** |
| --- | --- | --- |
| Anguilla | N/A |  |
| Argentina | 2 - 41.1% | [1, 2] |
| Aruba | N/A |  |
| Bahamas | 53.5% | [3] |
| Barbados | N/A |  |
| Belize | N/A |  |
| Bermuda | N/A |  |
| Bolivia | 33% | [4] |
| Brazil | 1 - 69% | [1, 2, 5, 6, 7] |
| Chile | N/A |  |
| Colombia | 4.4 - 10.82% | [1, 2, 8, 9] |
| Costa Rica | 3.5 - 22.5% | [10, 11, 12, 13, 14] |
| Cuba | 7 – 63% | [15, 16, 17, 18] |
| Dominican Republic | 18 - 18.2% | [19] |
| Ecuador | 0 – 36% | [20, 21, 22, 23] |
| El Salvador | 3-19% | [24, 25] |
| Grenada | 9.1-26.8%; | [26] |
| Guatemala | 29.7% | [27] |
| Guyana | 10% | [28] |
| Haiti | 26.2% | [29] |
| Honduras | 11.4 – 56% | [30, 31, 32] |
| Jamaica | 18.7% | [33] |
| Netherlands Antilles | 7.2 – 13.5% | [34, 35] |
| Nicaragua | 1.80% | [36] |
| Panama | 4% | [37] |
| Peru | 0 – 12.8% | [1, 38, 39, 40, 41, 42] |
| Puerto Rico | 6.7 – 20% | [43] |
| St. Kitts & Nevis | 7% | [44] |
| Suriname | 22% | [1] |
| Trinidad & Tobago | N/A |  |
| Turks & Caicos | 8 – 58% | [45] |
| Uruguay | N/A |  |
| U.S. Virgin Islands | N/A |  |
| Venezuela | 2 – 29% | [1, 46] |

**References**

1.	Bendas AJR, Mendes-de-Almeida F, Guerrero J, Labarthe N. Update on Dirofilaria immitis epidemiology in South America and Mexico: literature review. Brazilian Journal of Veterinary Research and Animal Science. 2017;54 4:319–29; doi: 10.11606/issn.1678-4456.bjvras.2017.132572.

2.	Labarthe N, Guerrero J. Epidemiology of heartworm: What is happening in South America and Mexico? Veterinary Parasitology. 2005;133 2:149–56; doi: 10.1016/j.vetpar.2005.04.006.

3.	Grieve RB, Glickman LT, Bater AK, Mika-Grieve M, Thomas CB, Patronek GJ. Canine Dirofilaria immitis infection in a hyperenzootic area: examination by parasitologic findings at necropsy and by two serodiagnostic methods. Am J Vet Res. 1986;47 2:329–32.

4.	Bronson E, Emmons LH, Murray S, Dubovi EJ, Deem SL. Serosurvey of Pathogens in Domestic Dogs on the Border of Noël Kempff Mercado National Park, Bolivia. Journal of Zoo and Wildlife Medicine. 2008;39 1:28–36, 9; doi: 10.1638/2006-0046.1.

5.	Chocobar MLE, Schmidt EMdS, Weir W, Panarese R. The Distribution, Diversity, and Control of Dirofilariosis in Brazil: A Comprehensive Review. Animals. 2024;14 17:2462; doi: 10.3390/ani14172462.

6.	Araújo TR, Lima PPdABMd, Paulino PG, Senne NA, Silva NMd, Souza LFd, et al. Epidemiology of Dirofilaria immitis and Acanthocheilonema reconditum in dogs from non-endemic municipalities at different altitudes in the State of Rio de Janeiro, Brazil. Research in Veterinary Science. 2025;196:105921; doi: 10.1016/j.rvsc.2025.105921.

7.	Félix NA, Neto ASdS, Rodrigues EMF, Leal-Lima A, Nunes-Pinheiro DCS, Ferreira TC. Seroprevalence of Dirofilaria immitis and assessment of systemic inflammatory indexes in apparently healthy dogs from coastal northeastern Brazil. Topics in Companion Animal Medicine. 2026;70:101028; doi: 10.1016/j.tcam.2025.101028.

8.	Esteban-Mendoza MV, Arcila-Quiceno V, Albarracín-Navas J, Hernández I, Flechas-Alarcón MC, Morchón R. Current Situation of the Presence of Dirofilaria immitis in Dogs and Humans in Bucaramanga, Colombia. Frontiers in Veterinary Science. 2020;7; doi: 10.3389/fvets.2020.00488.

9.	Muñoz AAF, Martinez AR, Pinilla JC. Prevalence of Dirofilaria immitis in shelter dogs in Bucaramanga metropolitan area, Colombia. Veterinary Parasitology: Regional Studies and Reports. 2020;22:100489; doi: 10.1016/j.vprsr.2020.100489.

10.	Montenegro VM, Bonilla MC, Kaminsky D, Romero-Zúñiga JJ, Siebert S, Krämer F. Serological detection of antibodies to Anaplasma spp., Borrelia burgdorferi sensu lato and Ehrlichia canis and of Dirofilaria immitis antigen in dogs from Costa Rica. Veterinary Parasitology. 2017;236:97–107; doi: 10.1016/j.vetpar.2017.02.009.

11.	Rojas A, Rojas D, Montenegro VM, Baneth G. Detection of Dirofilaria immitis and other arthropod-borne filarioids by an HRM real-time qPCR, blood-concentrating techniques and a serological assay in dogs from Costa Rica. Parasites & Vectors. 2015;8 1:170; doi: 10.1186/s13071-015-0783-8.

12.	Scorza AV, Duncan C, Miles L, Lappin MR. Prevalence of selected zoonotic and vector-borne agents in dogs and cats in Costa Rica. Veterinary Parasitology. 2011;183 1:178–83; doi: 10.1016/j.vetpar.2011.06.025.

13.	Wei L, Kelly P, Ackerson K, El-Mahallawy HS, Kaltenboeck B, Wang C. Molecular detection of Dirofilaria immitis, Hepatozoon canis, Babesia spp., Anaplasma platys and Ehrlichia canis in dogs on Costa Rica. Acta Parasitol. 2014;60 1:21–5; doi: 10.1515/ap-2015-0003.

14.	Springer A, Montenegro VM, Schicht S, Globokar Vrohvec M, Pantchev N, Balzer J, et al. Seroprevalence and Current Infections of Canine Vector-Borne Diseases in Costa Rica. Frontiers in Veterinary Science. 2019;6; doi: 10.3389/fvets.2019.00164.

15.	Duménigo R, Aguiar P, Galvez M. Prevalencia de Dirofilaria immitis en perros de Ciudad de la Habana. Rev Cubana Med Trop. 1982;34 3:262–8.

16.	Duménigo R, Espino A, Bouza M. Prevalencia de filariasis canina en la Isla de la Juventud. Rev Salud Anim. 1988;10:247–50.

17.	Pérez Martín O, Lastre González M, Aguiar PM, Gálvez M. Busqueda de Dirofilaria immitis en perros en la provincia Ciudad de La Habana. Rev cuba med trop. 1985:174–7.

18.	Sotolongo GF. Incidencia de la Dirofilaria immitis en los perros de la ciudad de La Habana. Rev Cubana Med Trop. 1977;29 1:9–12.

19.	Duran-Struuck R, Jost C, Hernandez AH. Dirofilaria immitis prevalence in a canine population in the Samana Peninsula (Dominican Republic) - June 2001. Vet Parasitol. 2005;133 4:323–7; doi: 10.1016/j.vetpar.2005.06.008.

20.	García Pontón JD: Recopilación de casos clínicos de la enfermedad Dirofilariósis (Dirofilaria immitis) Canina en el Ecuador. BABAHOYO: UTB, 2022; 2022.

21.	Levy JK, Crawford PC, Lappin MR, Dubovi EJ, Levy MG, Alleman R, et al. Infectious diseases of dogs and cats on Isabela Island, Galapagos. J Vet Intern Med. 2008;22 1:60–5; doi: 10.1111/j.1939-1676.2007.0034.x.

22.	López-Rauschenberg MK, Macías-Vera F, Zambrano-Chávez D, Fimia-Duarte R, Zambrano-Gavilanes MP. Presence of Dirofilaria immitis (Leidy, 1856) in dogs from a shelter in the Sucre Canton, Ecuador. 2023.

23.	McCown M, Monterroso VH. Zoonotic and Infectious Disease Surveillance in Ecuador: Ehrlichia canis, Anaplasma phagocytophilum, Borrelia burgdorferi, and Dirofilaria immitis Prevalence Rates in Canines. J Spec Oper Med. 2011;11:61–5; doi: 10.55460/9U3U-BCIZ.

24.	Alvarado Sorto JM, Orellana Menjívar SE, Pichinte Gálvez LA. Determinación de presencia del gusano del corazón (Dirofilaria immitis) en perros domésticos (Canis lupus familiaris) en El Puerto de La Libertad, Departamento de La Libertad y Suchitoto, Departamento de Cuscatlán, El Salvador. 2013.

25.	Conde Landaverde IM, Escobar Rodríguez AM, Gómez Vides WM. Determinación de la presencia del antígeno de Dirofilaria inmitis por inmunocromatografía en pacientes caninos de cinco clínicas veterinarias en la zona norte de San Salvador. 2005.

26.	Coomansingh C, Yabsley M, Wagner N, Pinckney R, Bhaiyat MI, Chikweto A, et al. Meta-analysis of the prevalence of Dirofilaria immitis in dogs from Grenada, West Indies. Int J Vet Med Res Rep. 2015;2015:429690; doi: 10.5171/2015.429690.

27.	Bader J, Ramos RAN, Otranto D, Dantas-Torres F. Vector-borne pathogens in dogs from Guatemala, Central America. Veterinary Parasitology: Regional Studies and Reports. 2020;22:100468; doi: 10.1016/j.vprsr.2020.100468.

28.	Milstein MS, Shaffer CA, Suse P, Marawanaru A, Heinrich DA, Larsen PA, et al. A mixed-methods approach to understanding domestic dog health and disease transmission risk in an indigenous reserve in Guyana, South America. PLoS neglected tropical diseases. 2022;16 6:e0010469; doi: 10.1371/journal.pntd.0010469.

29.	Starkey LA, Newton K, Brunker J, Crowdis K, Edourad EJP, Meneus P, et al. Prevalence of vector-borne pathogens in dogs from Haiti. Veterinary Parasitology. 2016;224:7–12; doi: 10.1016/j.vetpar.2016.04.017.

30.	Bodden Rosales GF: Determinación de la concordancia entre el método Knott y Snap 4Dx para diagnóstico de Dirofilaria immitis en perros. Universidad de San Carlos de Guatemala; 2023.

31.	Sánchez Alvarado MA: Determinación de la prevalencia de Dirofilaria immitis en perros del albergue de la Organización Rescatistas de Corazón, del municipio de San Pedro Sula, Cortés, Honduras en el año 2018. Universidad de San Carlos de Guatemala; 2019.

32.	Soto Castro SJ: Determinación de la incidencia de la Dirofilaria immitis por medio del método de Knott, en el municipio de Roatán, Islas de la Bahía. Universidad de San Carlos de Guatemala; 2007.

33.	Khouri NK, Singh S, Noble SA, Kirlew TV, Golding MA, Ali I, et al. Prevalence of Dirofilaria immitis in dogs in Jamaica. Parasitology Research. 2024;123 12:411; doi: 10.1007/s00436-024-08434-4.

34.	Hesselink JW. [The prevalence of heart worm (Dirofilaria immitis) in dogs of Curaçao]. Tijdschr Diergeneeskd. 1988;113 15-16:853–9.

35.	Saleh FC, Kirkpatrick CE, De Haseth O, Lok JB. Occurrence of some blood and intestinal parasites in dogs in Curaçao, Netherlands Antilles. Trop Geogr Med. 1988;40 4:318–21.

36.	Springer A, Montenegro VM, Schicht S, Pantchev N, Strube C. Seroprevalence and current infections of canine vector-borne diseases in Nicaragua. Parasites & Vectors. 2018;11 1:585; doi: 10.1186/s13071-018-3173-1.

37.	Chacón SC, de Candanedo PGC, Bermúdez S. Seroprevalence in Dogs Shows a High Incidence of Vector-Borne Diseases in Chiriquí Province, Panama. Journal of Veterinary Medicine and Animal Sciences. 2024;7 2:1149.

38.	Acuña P, Chávez A. Determinación de la prevalencia de Dirofilaria immitis en los distritos de San Martín de Porres, Rímac y Cercado de Lima. Revista de Investigaciones Veterinarias del Perú. 2002;13 2:108–10.

39.	Adrianzén J, Chávez A, Casas E, Li O. Seroprevalencia de la dirofilariosis y ehrlichiosis canina en tres distritos de Lima. Revista de Investigaciones Veterinarias del Perú. 2003;14 1:43–8.

40.	Bravo R, Chávez A, Casas E, Suárez F. Dirofilariosis canina en los distritos colindantes con la ribera del río Lurín. Revista de Investigaciones Veterinarias del Perú. 2002;13 1:80–3.

41.	Chipana C, Chávez A, Casas E, Suárez F. Estudio de la dirofilariosis canina en la ribera del río Chillón, Lima. Revista de Investigaciones Veterinarias del Perú. 2002;13 1:72–6.

42.	Corimanya J, Chávez A, Casas E, Díaz D. Frecuencia de Dirofilaria immitis en caninos del distrito de San Juan de Lurigancho. Revista de Investigaciones Veterinarias del Perú. 2004;15 2:141–4.

43.	McCown M, Opel T, Grzeszak B. Vector-Borne Disease Surveillance in Puerto Rico: Pathogen Prevalence Rates in Canines - Implications for Public Health and the U.S. Military - Applying the One Health Concept. Journal of special operations medicine : a peer reviewed journal for SOF medical professionals. 2013;13:59–63; doi: 10.55460/98QX-CJUU.

44.	Conan A, Napier P, Shell L, Knobel DL, Dundas J, Scorpio D, et al. Heterogeneous distribution of Dirofilaria immitis in dogs in St. Kitts, West Indies, 2014–2015. Veterinary Parasitology: Regional Studies and Reports. 2017;10:139–42; doi: 10.1016/j.vprsr.2017.10.006.

45.	Hoff B, McEwen B, Peregrine AS. A survey for infection with Dirofilaria immitis, Ehrlichia canis, Borrelia burgdorferi, and Babesia canis in feral and client-owned dogs in the Turks and Caicos Islands, British West Indies. Can Vet J. 2008;49 6:593–4.

46.	Guilarte DV, Gómez Martínez E, El Hen F, Guzmán R, Blondell D, Díaz MT, et al. Diagnóstico de Dirofilaria immitis en el municipio Sucre, estado Sucre, Venezuela. Boletín de Malariología y Salud Ambiental. 2011;51 1:51–8.
